# Chronological change of left ventricular global longitudinal strain in patients with maternally inherited diabetes and deafness: A case series

**DOI:** 10.1097/MD.0000000000037447

**Published:** 2024-03-08

**Authors:** Jeong-Sook Seo

**Affiliations:** a Division of Cardiology, Department of Internal Medicine, Busan Paik Hospital, Inje University, Busan, South Korea.

**Keywords:** cardiomyopathy, case report, echocardiography, longitudinal strain, m.3243A>G mutation, MIDD, mitochondrial disease

## Abstract

**Rationale:**

Maternally inherited diabetes and deafness (MIDD) is a rare genetic disorder arising from mitochondrial DNA mutations, characterized by a combination of diabetes mellitus and sensorineural deafness. It is known that MIDD patients with cardiomyopathy have a poor prognosis, but there are no established guidelines for the diagnosis and follow-up of cardiomyopathy in MIDD patients.

**Patient concerns:**

Patient 1 was a 48-year-old woman who visited the hospital with cardiomegaly and had been taking oral hypoglycemic agents for 8 years. Patient 2 was a 21-year-old man, the son of patient 1, who visited the hospital for genetic screening. Patient 2 was also diagnosed diabetes mellitus 2 years ago.

**Diagnosis:**

Patient 1 was found to have restrictive cardiomyopathy on echocardiography and underwent endomyocardial biopsy and genetic testing to determine the etiology. The m.3243A>G mutation was confirmed and she was diagnosed with MIDD accompanied with diabetes and hearing loss. Additionally, patient 2 had m.3243 A>G mutation and was diagnosed with MIDD due to diabetes and hearing loss.

**Interventions:**

Because MIDD does not have a specific treatment, patient 1 took ubidecarenone (coenzyme Q10), acetylcarnitine, and multivitamin along with the treatment for diabetes control and heart failure. Patient 2 was taking ubidecarenone (coenzyme Q10), acetylcarnitine, and multivitamin along with treatment for diabetes.

**Outcomes:**

She subsequently underwent routine transthoracic echocardiography, and a progressive decline in global longitudinal strain (GLS) was first observed, followed by a worsening of the patient’s clinical situation. Patient 2 had concentric remodeling and decreased GLS. On periodic echocardiography, GLS decreased at a very slow rate, and the patient’s clinical course was stable.

**Lessons:**

The findings of this report contribute to the understanding of the clinical course of MIDD-associated cardiomyopathy and highlight the potential of GLS as a sensitive marker for disease progression.

## 1. Introduction

Maternally inherited diabetes and deafness (MIDD) is a rare genetic disorder caused by a mitochondrial DNA mutation.^[1–5]^ It is characterized by a combination of diabetes mellitus and sensorineural deafness.^[6]^ MIDD can also affect other body parts, including the eyes, nervous system, and kidneys, and may cause cardiac dysfunction. Approximately 60% of MIDD patients may have some form of cardiac dysfunction, including cardiomyopathy and conduction abnormalities.^[7–10]^ Although it is important to detect cardiac involvement and monitor cardiac dysfunction in MIDD patients, data on what, how, and how often to follow-up are lacking. Thus, in this paper, 2 cases of MIDD with cardiomyopathy are described, detailing their clinical profiles, diagnostic findings, treatment approaches, and disease progression.

## 2. Case presentation

### 2.1. Patient 1

A 48-year-old woman visited our hospital for the evaluation of cardiomegaly detected by chest X-ray during a health checkup. She had been on oral glucose-lowering agents for 8 years because of diabetes mellitus. She denied symptoms, except for mild weakness of both lower extremities. During history taking, she demonstrated usual cognition and intelligence but often asked the doctor to repeat questions. Her height, weight, and body mass index were 151 cm, 34 kg, and 14.9 kg/m^2^, respectively. Her initial blood pressure was 100/60 mm Hg, and her pulse rate was 73 beats/minute.

**Figure 1.**
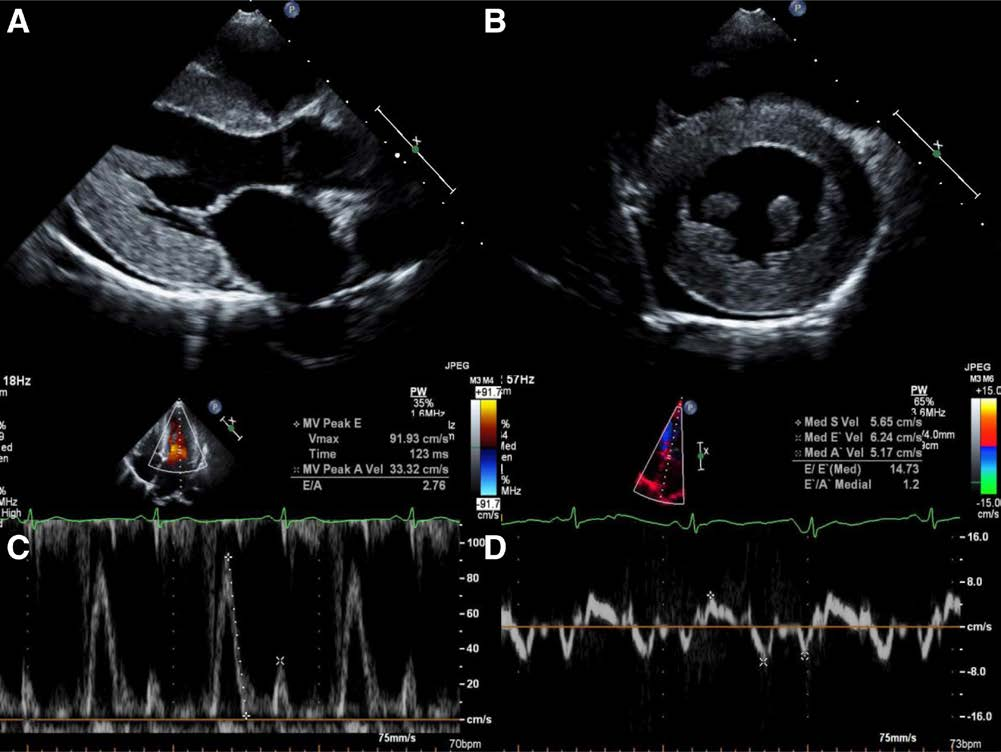
Transthoracic echocardiograms of Patient 1. (A) Parasternal long axis. (B) Parasternal short axis. (C) Mitral inflow. (D) Septal mitral annulus tissue Doppler image.

**Figure 2.**
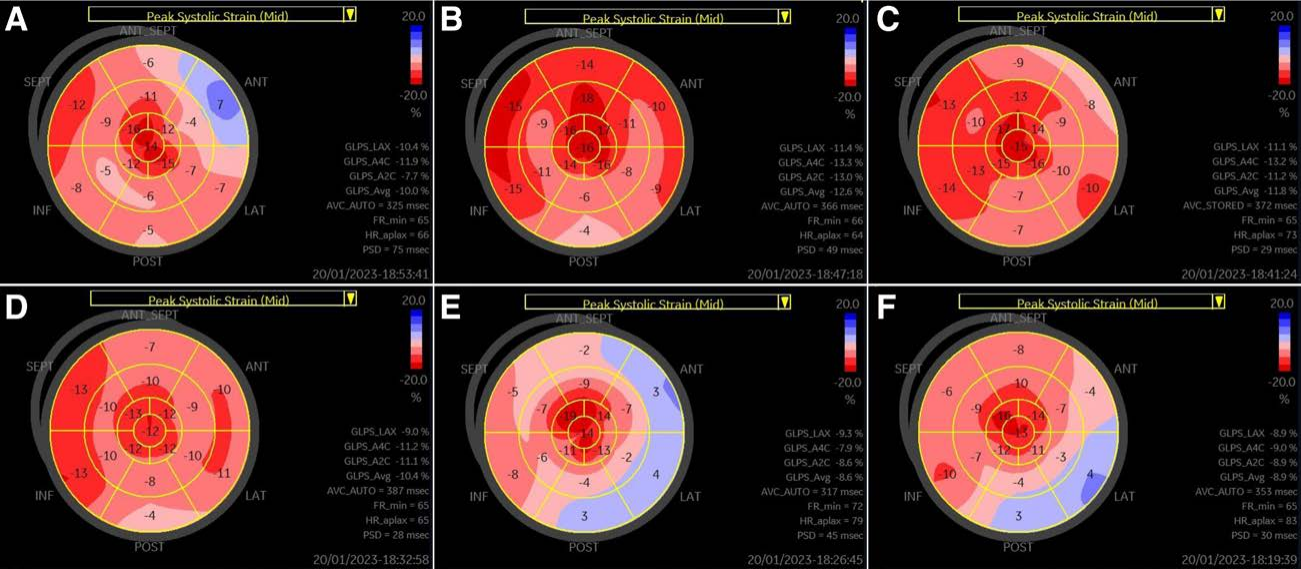
Chronological change of the global longitudinal strain of Patient 1. (A) Initial (B) 1-year follow-up. (C) Two-year follow-up. (D) Three-year follow-up. (E) Four-year follow-up. (F) Five-year follow-up.

The laboratory test results were as follows: markedly elevated serum N-terminal prohormone brain natriuretic peptide level at 1047.6 pg/mL; slightly elevated serum lactic acid level at 2.08 mmol/L; and mildly elevated creatine kinase-MB and troponin-I levels at 8.6 and 0.078 ng/mL, respectively. Electrocardiography revealed left ventricular hypertrophy (LVH) without conduction abnormalities. Chest radiography showed mild cardiomegaly without pulmonary edema. Severe concentric LVH (left ventricular mass index, 220.7 g/m^2^; relative wall thickness, 0.66) and restrictive physiology of diastolic filling (Fig. 1) were also noted by transthoracic echocardiography. Additionally, a small amount of pericardial effusion was seen. Although the left ventricular (LV) ejection fraction (EF) was preserved at 58%, global longitudinal strain (GLS) was decreased (−10.0%) (Table 1 and Fig. 2A). The longitudinal strain in the basal-to-mid anteroseptal, anterior, lateral, posterior, and inferior walls was also markedly reduced.

**Table 1:** Longitudinal change in cardiac structure and function in Patient 1.

Year (time lapse after diagnosis)	2015 (baseline)	2016 (1 year)	2017 (2 year)	2018 (3 year)	2019 (4 year)	2020 (5 year)
LV septal thickness, mm	14.2	14.3	14.4	15.1	16.1	15.0
LV posterior wall thickness, mm	14.9	14.1	14.4	14.6	14.6	16.0
LV end-diastolic dimension, mm	45.0	44.5	45.4	43.73	48.61	44.3
LV mass index, g/m^2^	216.0	235.3	247.2	208.1	268.7	205.0
LV EF, %	58.1	58.8	62.3	56.7	36.0	32.3
LV GLS, %	−10.0	−12.6	−11.8	−10.4	−8.6	−8.9
Mitral E velocity, cm/s	91.9	109.4	100.7	103.9	99.3	109.9
Mitral A velocity, cm/s	33.3	49.9	36.9	36.7	30.2	34.0
E’ velocity, cm/sec	6.2	6.87	6.9	6.7	5.8	5.7
E/e’ ratio	14.8	15.9	14.6	15.6	17.1	19.3
HbA1C, %	7.8	9.6	7.2	7.8	11.0	7.7
Creatinine, mg/dL	0.91	1.59	1.52	1.56	1.57	2.68
eGFR, mL/min/1.73m^2^	74.6	37.7	39.7	38.2	37.6	19.6
NT-proBNP, pg/mL	1047.6	538.6	736.3	593.3	21,088.4	12,442.5

**Figure 3.**
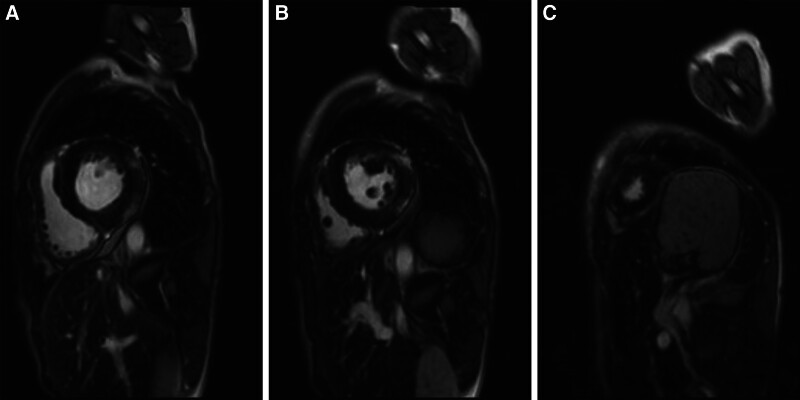
Delayed gadolinium enhancement of the cardiac magnetic resonance images of Patient 1. (A) Left ventricular (LV) base, (B) mid LV, and (C) LV apex.

Cardiac magnetic resonance imaging (MRI) was performed to differentiate myocardial diseases. On the fat-suppressed T2-weighted images, an increased signal intensity in the inferolateral and anterolateral regions, consistent with edema, was noted. A late gadolinium enhancement was seen in the basal-to-mid inferolateral and anterolateral walls of the LV subepicardial myocardium (Fig. 3).

**Figure 4.**
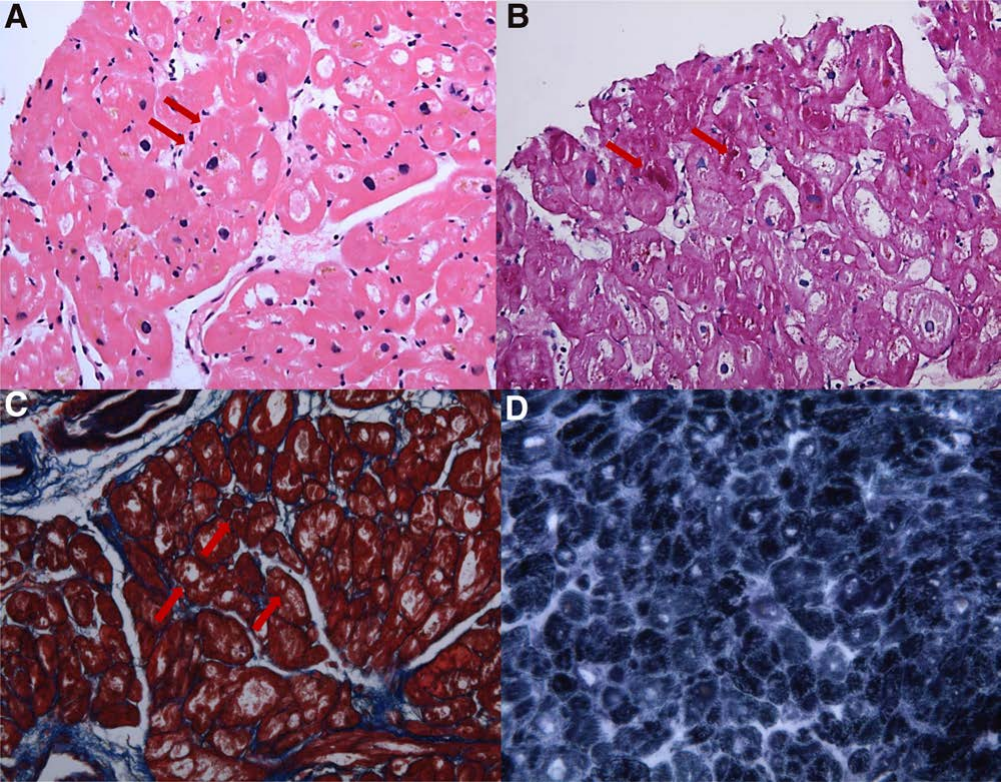
Microscopic findings of Patient 1. (A) Hematoxylin and eosin staining, magnification ×400. (B) Periodic acid–Schiff staining, magnification ×400. (C) Masson trichrome staining, magnification ×400. (D) Nicotinamide adenine dinucleotide staining, magnification ×400.

**Figure 5.**
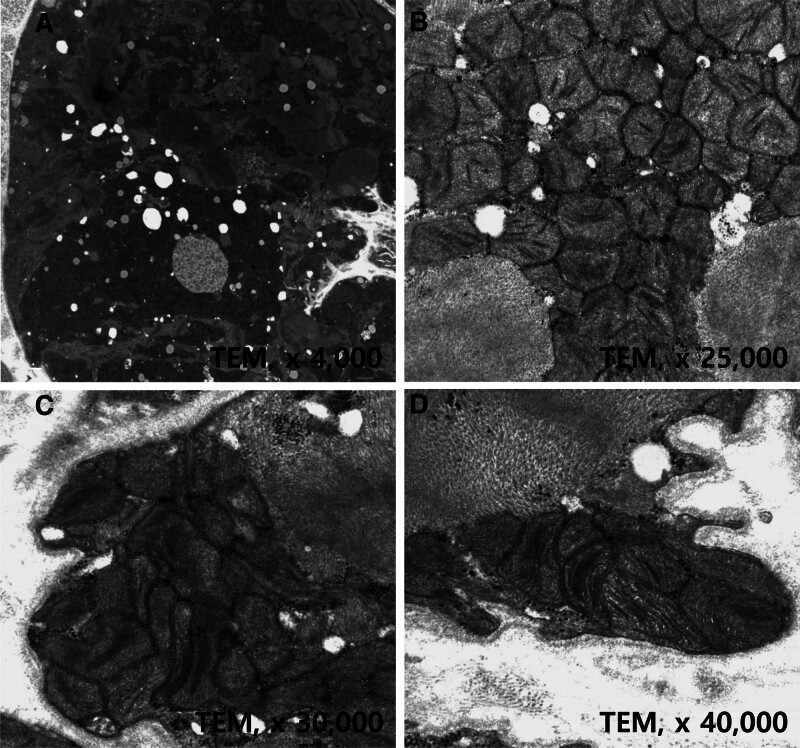
Transmission electron microscopic findings of Patient 1. (A) Magnification ×4000. (B) Magnification ×25,000. (C) Magnification ×30,000. (D) Magnification ×40,000.

Cardiac myocytes showed diffuse vacuolar degeneration, central nuclei, increased pigmentation, and irregular cell borders with hematoxylin and eosin staining (Fig. 4A). Periodic acid–Schiff (PAS) staining showed irregularly accumulated PAS-positive materials in the subsarcolemmal and intracytoplasmic areas (Fig. 4B). Myocytes with irregular cell borders appeared as ragged-red fibers with PAS, Masson trichrome, and nicotinamide adenine dinucleotide staining (Fig. 4B–D). A Congo red test showed negative findings. These histologic findings suggested the presence of a mitochondrial disease. To evaluate the nature of the ragged-red myofibers, transmission electron microscopy was performed, which demonstrated an accumulation of abnormal-shaped mitochondria of varying sizes in the subsarcolemmal area and center of the myocytes (Fig. 5A). Electron-dense inclusions and thickened and disoriented cristaes were found in the abnormal mitochondria (Fig. 5B and C), which sometimes formed concentric lamelli (Fig. 5D).

Electromyography, which was performed to evaluate the weakness of her lower extremities, revealed findings suggestive of myopathy of both lower extremities. Additionally, the patient was diagnosed with sensorineural hearing loss by pure-tone audiometry, which was performed because hearing loss was suspected during medical history taking.

Based on the abovementioned findings, the patient was suspected to have mitochondrial disease. A sequence analysis of the mitochondrial DNA revealed heteroplasmy of m.3243A>G (rs199474657) and wild type. She had no history of stroke or seizure, and no evidence of ischemia or bleeding on brain MRI. Finally, she was diagnosed with MIDD with cardiomyopathy and was treated with captopril, furosemide, spironolactone, ubidecarenone (coenzyme Q10), acetylcarnitine, and multivitamin.

Table 1 shows Patient 1’s echocardiographic parameters and laboratory results in chronological order. During the first year, the patient’s LV EF and LV wall thickness did not change, and her GLS improved (−12.6%). Over the next 2 years, she remained well without worsening of symptoms and there were no LV EF and LV wall thickness changes, but her GLS gradually decreased. Echocardiography performed at 4 years after diagnosis showed a significant decrease in the LV EF to 36% and GLS to − 8.6%, but the LV wall thickness did not change. At this time, the N-terminal prohormone brain natriuretic peptide (NT-pro BNP) level was markedly increased to 21,088.4 pg/mL. From this point on, the patient was hospitalized once a year due to worsening symptoms, such as dyspnea and generalized edema. Despite receiving oral hypoglycemic agents and insulin treatment under the supervision of a specialist, the patient’s glucose level was poorly controlled during the follow-up period, and the patient’s poor compliance was considered one of the causes. Additionally, the patient’s renal function also gradually declined during the follow-up period.

### 2.2. Patient 2

Patient 2 was the 21-year-old son of Patient 1. On family gene screening, heteroplasmy of m.3243 A>G was confirmed. His height, weight, and body mass index were 163 cm, 53 kg, and 19.95 kg/m^2^, respectively. He also had been on oral glucose-lowering agents, including linagliptin, glimepiride, and metformin, for 2 years.

**Figure 6.**
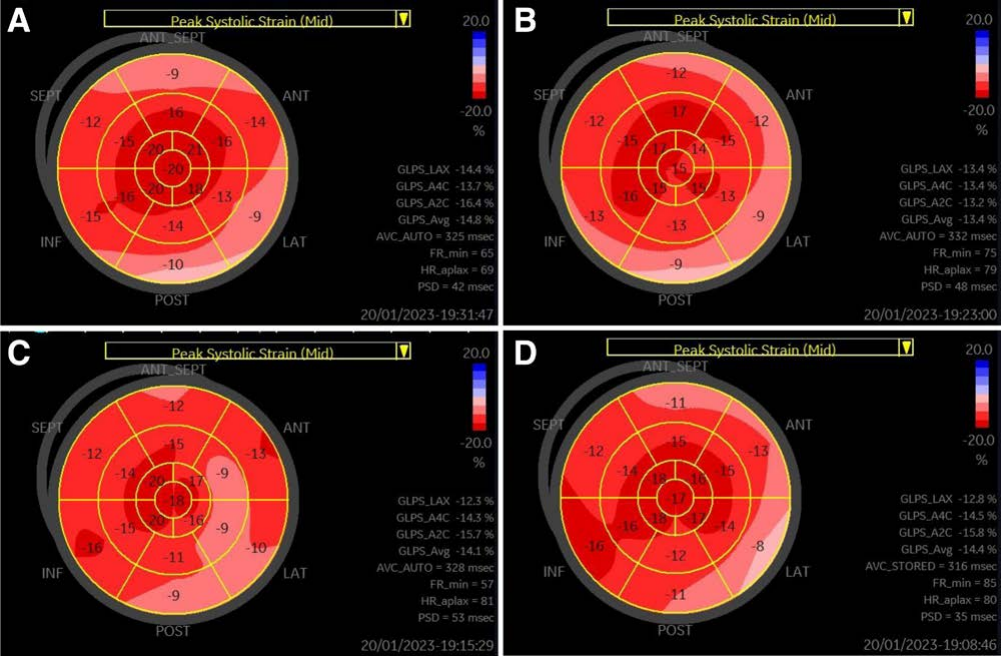
Chronological change of the global longitudinal strain of Patient 2. (A) Initial. (B) 2-year follow-up. (C) Four-year follow-up. (D) Six-year follow-up.

Electrocardiogram showed sinus arrhythmia and LVH by voltage. In transthoracic echocardiography, the LV septum and posterior wall thicknesses were 11.2 and 10.67 mm, respectively, and the LV mass index was 103.98 g/m^2^. The relative wall thickness was 0.49, confirming concentric remodeling. The GLS value of LV was −14.8% (Fig. 6A). The longitudinal strain values of the basal anteroseptum, lateral, and posterior segments were relatively lower. Cardiac MRI did not show a late gadolinium enhancement.

While conducting tests to check for combined abnormalities, the patient was diagnosed with bilateral sensorineural hearing loss by pure-tone audiometry. Although he did not complain of muscle weakness, the electromyography findings suggested myopathy.

He was diagnosed with MIDD with cardiomyopathy and was treated with ubidecarenone, acetylcarnitine, and multivitamin.

For the 6-year follow-up period, the patient’s symptoms have been stable. However, audiometry showed the progression of sensory neural deafness (pure tone Rt 38 → 45dB, Lt 38 → 41dB). The changes in the echocardiographic parameters and laboratory results of Patient 2 are summarized in Table 2. The patient’s glucose control showed gradual deterioration. At the time of MIDD diagnosis, the hemoglobin A1C level was well controlled at 6.1% while taking oral hypoglycemic agents, but since then, glucose control has gradually deteriorated and insulin was administered along with oral hypoglycemic agents. The patient’s TTE was monitored every 2 years, the LV wall thickness and mass index gradually decreased, and no findings suggestive of LVH were observed. The LV EF was also preserved, but it slightly decreased compared to that obtained during the initial examination. The LV GLS was slightly decreased from the time of diagnosis, but no additional decreases were observed during the follow-up period (Table 2 and Fig. 6). The N-terminal prohormone brain natriuretic peptide levels were also maintained at normal levels.

**Table 2:** Longitudinal change in cardiac structure and function in Patient 2.

Year (time lapse after diagnosis)	2016 (baseline)	2018 (2 year)	2020 (4 year)	2022 (6 year)
LV septal thickness, mm	11.2	10.9	8.4	8.3
LV posterior wall thickness, mm	10.7	10.5	8.4	8.7
LV end-diastolic dimension, mm	43.0	44.8	45.2	39.6
LV mass index, g/m^2^	105.3	106.5	78.1	66.2
LV EF, %	60.9	64.3	56.6	51.2
LV GLS, %	−14.8	−13.4	−14.1	−14.4
Mitral E velocity, cm/s	81.2	77.2	91.34	63.2
Mitral A velocity, cm/s	53.7	55.9	58.03	53.6
E’ velocity, cm/s	11.6	12.3	13.0	7.8
E/e’ ratio	7.0	6.3	7.0	8.1
HbA1C, %	6.1	6.9	7.7	8.2
Creatinine, mg/dL	1.02	1.13	0.99	1.04
eGFR, mL/min/1.73 m^2^	104.6	91.1	105.4	97.9
NT-proBNP, pg/mL	26.7	16.9	19.0	15.3

## 3. Discussion and conclusion

This case report presents 2 cases of MIDD with cardiomyopathy. Particularly, the changes in cardiac functioning including LV GLS over time by performing periodic follow-up TTE were detailed. The LV GLS in the basal anterior, lateral, and posterior segments of the 2 patients were particularly impaired, which was similar to the LGE pattern of cardiac MRI in Patient 1 and was observed still in Patient 2, despite the absence of LGE. In both patients, the LV GLS was decreased even when EF was normal, and the decrease in GLS preceded the decrease in EF. Particularly, in Patient 2, GLS was decreased despite the presence of normal LV wall thickness and normal EF.

MIDD is caused by a point mutation in mitochondrial DNA and is transmitted in a maternal mode. The most common mutation is m.3243A>G; however, point mutations at 568 and 8281 have been identified in rare cases.^[1–5]^ MIDD accounts for 30% of the syndromic phenotypic spectrum of m.3243A>G and, as its name presents, is characterized by diabetes mellitus and deafness.^[6]^ It differs from the mitochondrial encephalomyopathy, lactic acidosis, and stroke-like episodes syndrome by the absence of neurological symptoms, including stroke-like episode and encephalopathy.^[11]^

The most common cardiac manifestation is LVH, which is observed in 55% of MIDD patients.^[7,10]^ Cardiomyopathy is also found in 15% to 30% of MIDD patients and cardiomyopathy can appear as both hypertrophic and dilated cardiomyopathies.^[9,10]^ Approximately 27% of patients may have conduction disturbance, and the most common arrhythmias are Wolff–Parkinson–White syndrome, sick sinus syndrome, and atrial fibrillation.^[8–10]^

Although accurate data on mortality in MIDD patients with cardiomyopathy are lacking, patients with m.3243A>G mutation are known to have a higher mortality rate than patients without cardiomyopathy in both adults and children with cardiomyopathy.^[12,13]^ Additionally, approximately 1/3 of deaths are caused by neurological and cardiovascular causes, with sudden and unexpected deaths accounting for a significant portion of them. Therefore, early detection of cardiomyopathy and progression monitoring in MIDD patients are very important for predicting and managing the patient’s prognosis. Some experts recommend that MIDD patients begin cardiac screening by undergoing electrocardiogram and echocardiogram at ≤35 years of age, and then annual cardiac evaluation and an echocardiogram every 3 to 5 years. However, because the rate of cardiomyopathy progression varies depending on the patient’s degree of heteroplasmy and control of concomitant diseases, the optimal period and method of follow-up are difficult to determine.

There are a few studies that have screened cardiac function using 2D speckle-tracking echocardiography in patients with MIDD or mitochondrial disease.^[14–16]^ However, in most cases, only one test was performed at the initial diagnosis. A retrospective study showed changes in GLS values in patients who underwent echocardiography more than twice, but the follow-up periods differed for all patients.^[14]^ Additionally, the study did not include all LV segments because it used an apical 4 chamber and mid-cavity short-axis view, unlike standard GLS analysis guidelines. In contrast, this report has the strengths of regularly monitoring cardiac structure and function, including the conventional parameters of LV dimension, volume, mass index, and EF, especially GLS, an early marker of systolic dysfunction.

In conclusion, the findings of this report suggest that GLS has the potential to provide additional benefits not only in early diagnosis but also in follow-up of cardiomyopathy in MIDD patients. The changes in GLS preceded the changes in EF or LV structure, which were classically used to evaluate LV function, and were found to fit well with clinical prognosis. Monitoring the chronological change in GLS can provide valuable information about the rate of decline, response to treatment, and overall prognosis. It allows clinicians to tailor their management strategies and timely interventions based on the specific changes observed in each patient to optimize patient outcomes. Although further studies are needed, this report suggests that GLS may be a better indicator for cardiac monitoring in MIDD patients.

## Acknowledgments

The author would like to thank Enago (www.enago.co.kr) for the English language review.

## Author contributions

**Conceptualization:** Jeong-Sook Seo.

**Data curation:** Jeong-Sook Seo.

**Formal analysis:** Jeong-Sook Seo.

**Investigation:** Jeong-Sook Seo.

**Methodology:** Jeong-Sook Seo.

**Project administration:** Jeong-Sook Seo.

**Resources:** Jeong-Sook Seo.

**Validation:** Jeong-Sook Seo.

**Visualization:** Jeong-Sook Seo.

**Writing – original draft:** Jeong-Sook Seo.

**Writing – review & editing:** Jeong-Sook Seo.

## References

[1] Maassen JA Janssen GM t Hart LM. Molecular mechanisms of mitochondrial diabetes (MIDD). Ann Med. 2005;37:213–21. 16019720 10.1080/07853890510007188

[2] Chae JH Hwang H Lim BC. Clinical features of A3243G mitochondrial tRNA mutation. Brain Dev. 2004;26:459–62. 15351082 10.1016/j.braindev.2004.01.002

[3] Hansrote S Croul S Selak M. External ophthalmoplegia with severe progressive multiorgan involvement associated with the mtDNA A3243G mutation. J Neurol Sci. 2002;197:63–7. 11997068 10.1016/s0022-510x(02)00048-5

[4] Verma A Moraes CT Shebert RT. A MERRF/PEO overlap syndrome associated with the mitochondrial DNA 3243 mutation. Neurology. 1996;46:1334–6. 8628477 10.1212/wnl.46.5.1334

[5] Goto Y Nonaka I Horai S. A mutation in the tRNA(Leu) (UUR) gene associated with the MELAS subgroup of mitochondrial encephalomyopathies. Nature. 1990;348:651–3. 2102678 10.1038/348651a0

[6] Nesbitt V Pitceathly RDS Turnbull DM. The UK MRC Mitochondrial Disease Patient Cohort Study: clinical phenotypes associated with the m.3243A>G mutation-implications for diagnosis and management. J Neurol Neurosurg Psychiatry. 2013;84:936–8. 23355809 10.1136/jnnp-2012-303528

[7] Majamaa-Voltti K Peuhkurinen K Kortelainen M-L. Cardiac abnormalities in patients with mitochondrial DNA mutation 3243A>G. BMC Cardiovasc Disord. 2002;2:12. 12150714 10.1186/1471-2261-2-12 PMC119851

[8] Malfatti E Laforêt P Jardel C. High risk of severe cardiac adverse events in patients with mitochondrial m.3243A>G mutation. Neurology. 2013;80:100–5. 23243073 10.1212/WNL.0b013e31827b1a2f

[9] Niedermayr K Pölzl G Scholl-Bürgi S. Mitochondrial DNA mutation “m.3243A>G”—heterogeneous clinical picture for cardiologists (“m.3243A>G”: a phenotypic chameleon). Congenit Heart Dis. 2018;13:671–7. 30133155 10.1111/chd.12634

[10] Duran J Martinez A Adler E. Cardiovascular manifestations of mitochondrial disease. Biology (Basel). 2019;8:34. 31083569 10.3390/biology8020034 PMC6628328

[11] Shen X Du A. The non-syndromic clinical spectrums of mtDNA 3243A>G mutation. Neurosciences (Riyadh). 2021;26:128–33. 33814365 10.17712/nsj.2021.2.20200145 PMC8024137

[12] Holmgren D Wåhlander H Eriksson BO. Cardiomyopathy in children with mitochondrial disease; clinical course and cardiological findings. Eur Heart J. 2003;24:280–8. 12590906 10.1016/s0195-668x(02)00387-1

[13] Ng YS Grady JP Lax NZ. Sudden adult death syndrome in m.3243A>G-related mitochondrial disease: an unrecognized clinical entity in young, asymptomatic adults. Eur Heart J. 2016;37:2552–9. 26188002 10.1093/eurheartj/ehv306 PMC5008417

[14] Koene S Timmermans J Weijers G. Is 2D speckle tracking echocardiography useful for detecting and monitoring myocardial dysfunction in adult m.3243A>G carriers? – a retrospective pilot study. J Inherit Metab Dis. 2017;40:247–59. 28054208 10.1007/s10545-016-0001-7 PMC5306433

[15] Hendrix CLF van den Heuvel FMA Rodwell L. Screening and prevalence of cardiac abnormalities on electro- and echocardiography in a large cohort of patients with mitochondrial disease. Mol Genet Metab. 2022;136:219–25. 35659503 10.1016/j.ymgme.2022.05.004

[16] Seiler F Ruile P Moser M. Bilateral deafness, diabetes, and different types of cardiomyopathy in family members with m.3243A > g mutation: a case report. Eur Heart J Case Rep. 2023;7:ytad073. 36865084 10.1093/ehjcr/ytad073 PMC9972186

